# Levonorgestrel-releasing intrauterine system placement for severe uterine cervical stenosis after conization: two case reports

**DOI:** 10.1186/s13256-016-0831-9

**Published:** 2016-03-09

**Authors:** Emi Motegi, Kiyoshi Hasegawa, Satoshi Kawai, Kaori Kiuchi, Nobuaki Kosaka, Yoshiko Mochizuki, Ichio Fukasawa

**Affiliations:** 1 Department of Obstetrics and Gynecology, Dokkyo Medical University, 880 Kitakobayashi, Mibu, Tochigi 321-0293 Japan; 2 Department of Obstetrics and Gynecology, Fujita Health University School of Medicine, Toyoake, Aichi 470-1192 Japan

**Keywords:** Uterine cervical stenosis, Cervical conization, Cervical intraepithelial neoplasia (CIN), Levonorgestrel- releasing intrauterine system (LNG-IUS)

## Abstract

**Background:**

Several approaches for treating severe uterine cervical stenosis after conization for cervical intraepithelial neoplasia have been reported; yet, the condition can still be difficult to treat successfully.

**Case presentation:**

We performed uterine cervical dilation surgery in two patients with severe stenosis, followed by insertion of the levonorgestrel-releasing intrauterine system, which is used for dysmenorrhea or endometriosis-related pain because of its strong progesterone activity. Patient 1 was a 34-year-old Japanese woman who was diagnosed with dysmenorrhea caused by recurrent uterine cervical stenosis and hematometra after laser conization. Patient 2 was a 44-year-old Japanese woman who developed dysmenorrhea and prolonged menstruation caused by uterine cervical stenosis without hematometra. After providing informed consent, they underwent cervical dilation surgery followed by insertion of the levonorgestrel-releasing intrauterine system. After treatment, their symptoms immediately improved, and after removal of their devices, they remained asymptomatic.

**Conclusions:**

To the best of our knowledge, this is the first report to confirm the usefulness and easy applicability of the levonorgestrel-releasing intrauterine system for uterine cervical stenosis. Although we had success with the method, this study of two patients is preliminary. Further study with larger numbers of patients is necessary to confirm the usefulness of our technique.

## Background

Uterine cervical conization is widely performed as both a diagnostic and a therapeutic procedure for patients with cervical intraepithelial neoplasia (CIN) or early invasive cervical carcinoma. The intra- and postoperative complications of uterine cervical conization include hemorrhage; cervical stenosis and occlusion; infection; cervical incompetence, which may lead to miscarriage or preterm birth during the second and third trimesters; and rare events such as uterine perforation, bladder or rectum injury, and pelvic inflammation [1–5].

Postprocedural uterine cervical stenosis is associated with menstrual disorders, hematometra, and infertility. The condition is also a concern because it prevents both adequate cytological follow-up and collection of endometrial cytology for carcinoma screening. The incidence of cervical stenosis after conization is reportedly 4 % to 17 % [6–11]. The occurrence of complete occlusion combined with hematometra is a rare but serious event, as it can be difficult to resolve [6, 7].

Several approaches for treating severe uterine cervical stenosis have been investigated: cervical dilation, cervicoplasty, cervical stent placement, and intrauterine device (IUD) insertion [12–17]. The treatment is determined according to the severity of stenosis or symptoms. The application of an IUD for cervical stenosis [14, 17] might be effective because the dilation required for device insertion allows menstrual blood to drain. The levonorgestrel-releasing intrauterine system (LNG-IUS) (Mirena®; Bayer Schering Pharma Oy, Espoo, Finland) is used not only for contraception but also for dysmenorrhea- or endometriosis-related pain [18]. The LNG-IUS exerts strong progesterone activity, which leads to profound thinning and atrophy of the endometrium. Estrogen receptors are suppressed during LNG use, also contributing to reduced menstrual bleeding. Therefore, LNG-IUS insertion may be expected to be effective for severe cervical stenosis with dysmenorrhea because of the sustained dilation of stenotic tissue with easy drainage of menstrual blood [14, 17], or because of the direct effect of progesterone on the endometrium.

We describe use of the LNG-IUS after cervical dilation surgery for two patients with severe stenosis after uterine cervical conization.

## Case presentations

### Patient 1

**Fig. 1:**
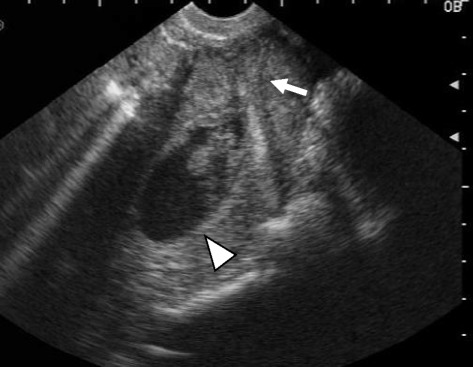
Preoperative ultrasound of patient 1. Hematometra is shown. *Arrow* and *arrowhead* represent cervix and hematometra, respectively

**Fig. 2:**
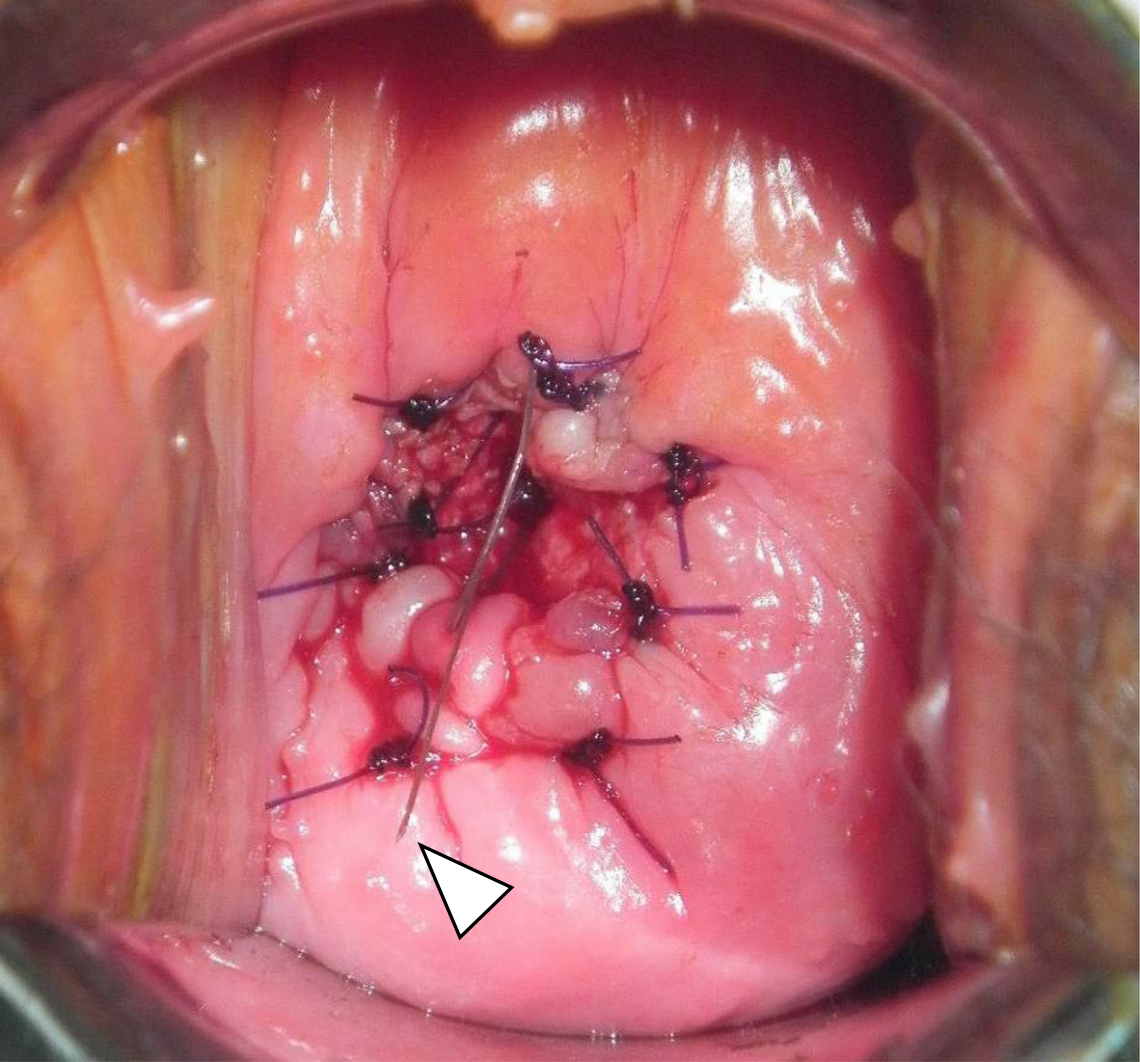
Post–cervical dilation surgery insertion of the levonorgestrel-releasing intrauterine system (LNG-IUS) in patient 1. Patient 1 underwent cervical dilation surgery followed by insertion of the LNG-IUS, The *arrowhead* represents a tail of the LNG-IUS. A cruciate incision was used at the external ostium. After dilation using the Hegar method, 3-0 polydioxanone monofilament sutures were used at eight points to evert the endocervical mucosa to the ectocervix

Patient 1 was a 34-year-old, gravida 0 para 0, Japanese woman with a diagnosis of uterine cervical microinvasive squamous cell carcinoma, International Federation of Gynecology and Obstetrics stage Ia1. Her past history and familial history were unremarkable. She underwent laser conization using a potassium titanyl phosphate/neodymium-doped yttrium aluminum garnet laser (Table 1). A 20-mm-long tissue cone was removed, with negative surgical margins. Seven months later, the patient developed severe abdominal cramping with prolonged but scant menstruation. She was diagnosed with dysmenorrhea caused by uterine cervical stenosis and hematometra. She underwent cervical dilation surgery while under spinal anesthesia. A cruciate incision was used at the external ostium, and the hematometra was evacuated. Hegar dilators were then employed, and 3-0 polydioxanone monofilament sutures (PDS II®; Ethicon Inc., Somerville, NJ, USA) were used at eight points to evert the endocervical mucosa to the ectocervix. The patient’s symptoms immediately improved, and her Numeric Rating Scale (NRS) [19] for menstruation decreased from 10 to 3 after treatment. However, she developed the same symptoms again 4 months later, with a NRS of 10, and was found to have recurrent hematometra (Fig. 1). After she provided informed consent, she again underwent cervical dilation surgery followed by insertion of the LNG-IUS (Fig. 2). After treatment, her symptoms again immediately improved, and the LNG-IUS was left in place for 5 months after treatment. Twenty months after removal of the LNG-IUS, she remained asymptomatic and her NRS for menstruation remained at 2.

**Table 1:** Patient profiles

	Patient 1	Patient 2
Age, years	34	44
Gravida and para status	0G0P	0G0P
Pathological diagnosis	Cervical carcinoma SCC, FIGO stage Ia1	Carcinoma *in situ*
Length of the cone removed	20 mm	16 mm
Symptoms	Dysmenorrhea, hematometra	Dysmenorrhea
Interval from conization to stenosis, months	7	13
Treatment	First: cervical dilation surgery Second: cervical dilatation surgery and insertion of the LNG-IUS	Cervical dilation surgery and insertion of the LNG-IUS
Outcome	No recurrence of stenosis for 20 months	No recurrence of stenosis for 12 months

*FIGO* International Federation of Gynecology and Obstetrics, *SCC* squamous cell carcinoma, *LNG-IUS* Levonorgestrel- releasing intrauterine system

### Patient 2

Patient 2 was a 44-year-old, gravida 0 para 0, Japanese woman with a diagnosis of carcinoma *in situ* of the uterine cervix. Her past history and familial history were unremarkable. She underwent laser conization with use of the holmium yttrium aluminum garnet laser (Table 1). A 16-mm-long tissue cone was removed, with negative surgical margins. Thirteen months later, she developed dysmenorrhea and prolonged menstruation caused by uterine cervical stenosis without hematometra. After providing informed consent, she underwent the same cervical dilation procedure as patient 1, followed by insertion of the LNG-IUS. Her symptoms improved, and the LNG-IUS was removed after 5 months. Twelve months later, she remained asymptomatic and her NRS for menstruation decreased from 10 (preprocedure) to 2.

### Summary

Neither patient experienced abnormal bleeding or any other symptoms associated with LNG-IUS use, including nausea, headache, edema, breast tenderness, weight gain, acne, or depressed mood.

## Discussion

Uterine cervical conization is a definitive treatment for patients with CIN, preserving the uterus while removing any invasion or residual lesions. Uterine cervical stenosis is not a rare complication of the procedure; however, its severity varies from patient to patient. There are several definitions of cervical stenosis with no consensus in the literature. Therefore, the reported incidence of cervical stenosis after conization varies from 4 % to 17 % [6–11].

Several studies have investigated the risk factors associated with uterine cervical stenosis after conization [8, 10, 20, 21]. Suh-Burgmann *et al*. reported a 6 % rate of cervical stenosis after loop electrosurgical excision procedure (LEEP) and that both the volume of tissue removed and a history of previous LEEP are significant independent predictors of stenosis [20]. Baldauf *et al*. reported a 4.3 % rate of cervical stenosis after conization, and in univariate analysis they found that the significant risk factors are age older than 50 years, an exclusively endocervical lesion, excision depth 20 mm or greater, and laser conization. Their multivariate analysis revealed excision depth and endocervical location as the only independent risk factors [8]. Houlard *et al*. reported that cervical stenosis after laser conization occurs in 16.8 % of patients, with patient age being the only independent risk factor [10]. CIN lesions sometimes extend from the ectocervix to the endocervix, even in premenopausal patients. It is therefore necessary to perform a sufficiently deep excision to avoid positive endocervical margins. However, this practice might increase the risk for cervical stenosis after conization.

Both of our patients underwent laser conization. In patient 1, it is possible that cervical stenosis resulted from the 20-mm incision needed for complete excision of her cervical lesion. In patient 2, the risk factors for cervical stenosis were her age and the endocervical location of the CIN lesion.

Several approaches to severe cervical stenosis have been reported. Dilation of the stenotic cervix is a relatively simple outpatient procedure; however, it must be performed several times to obtain optimal results. Holmskov *et al*. reported the usefulness of Hegar cervical dilators [15]. Luesley *et al*. reported the favorable results of carbon dioxide laser vaporization to remove scar tissue after conization in patients with symptomatic cervical stenosis [16]. Several reports have described successful treatment of severe cervical stenosis using different types of indwelling cervical stents to prevent restenosis after dilation. Something as simple as a urinary catheter [12] may be used, and Grund *et al*. reported that insertion of a nitinol stent appears to be a valid method of treating recurrent cervical stenosis and hematometra [13]. Whereas this self-expanding stent may be a useful device for severe stenosis, it is very expensive for routine use.

The literature contains some case reports on the use of IUDs for cervical stenosis. Puzey *et al*. reported success with a copper IUD [17], and Nasu *et al*. reported using nylon threads tied to an IUD and protruding through the stenotic cervical canal [14], providing a constitutive dilation force on the fibrous stenotic tissue and allowing drainage of menstrual blood and uterine fluid [14].

The choice of procedure or device is based on the severity of stenosis or the symptoms of the individual. A clinical dilemma arises when these more conservative methods fail. Hysterectomy is a method of last resort, particularly when patients wish to preserve fertility [13].

We report the usefulness of LNG-IUS insertion after cervical dilation surgery in two patients with uterine cervical stenosis. We believe that the effectiveness of this method is due to the constitutive dilation of stenotic cervical tissue, the ability for constant drainage of menstrual blood, and the direct effect of progesterone on the endometrium, reducing menstrual bleeding and pain. It is possible that cervical dilation alone might be effective for severe cervical stenosis; however, patient 1 in our series developed recurrent stenosis within a relatively short interval after cervical dilation. Therefore, we thought that the addition of LNG-IUS insertion would be an effective approach.

We left the LNG-IUS in place for 5 months; it would be interesting to see the results when the LNG-IUS is used for longer periods. It will also be interesting to see further study of the method of tying nylon threads to the LNG-IUS as previously reported [14]. This handmade device may be more effective than the LNG-IUS alone because it provides more constitutive dilation of the stenotic cervix along with the progesterone activity inherent to the LNG-IUS itself.

## Conclusions

This report is the first to confirm the usefulness of the LNG-IUS for uterine cervical stenosis. Although the device is easy to use and we had success with the method, this study of two patients is preliminary. Further study with larger numbers of patients is necessary to confirm the usefulness of our technique.

## Consent

Written informed consent was obtained from the patients for publication of this case report and any accompanying images. Copies of the written consents are available for review by the Editor-in-Chief of this journal.

## Abbreviations

CIN: cervical intraepithelial neoplasia
FIGO: International Federation of Gynecology and Obstetrics
IUD: intrauterine device
LEEP: loop electrosurgical excision procedure
LNG-IUS: levonorgestrel-releasing intrauterine system
NRS: Numeric Rating Scale
SCC: squamous cell carcinoma
